# Towards high-power mid-IR light source tunable from 3.8 to 4.5 µm by HBr-filled hollow-core silica fibres

**DOI:** 10.1038/s41377-021-00703-6

**Published:** 2022-01-13

**Authors:** Zhiyue Zhou, Zefeng Wang, Wei Huang, Yulong Cui, Hao Li, Meng Wang, Xiaoming Xi, Shoufei Gao, Yingying Wang

**Affiliations:** 1 College of Advanced Interdisciplinary Studies, National University of Defense Technology, Changsha, 410073 China; 2 State Key Laboratory of Pulsed Power Laser Technology, Changsha, 410073 China; 3 Hunan Provincial Key Laboratory of High Energy Laser Technology, Changsha, 410073 China; 4 Institute of Photonics Technology, Jinan University, Guangzhou, 511443 China

**Keywords:** Fibre lasers, Mid-infrared photonics

## Abstract

Fibre lasers operating at the mid-IR have attracted enormous interest due to the plethora of applications in defence, security, medicine, and so on. However, no continuous-wave (CW) fibre lasers beyond 4 μm based on rare-earth-doped fibres have been demonstrated thus far. Here, we report efficient mid-IR laser emission from HBr-filled silica hollow-core fibres (HCFs) for the first time. By pumping with a self-developed thulium-doped fibre amplifier seeded by several diode lasers over the range of 1940–1983 nm, narrow linewidth mid-IR emission from 3810 to 4496 nm has been achieved with a maximum laser power of about 500 mW and a slope efficiency of approximately 18%. To the best of our knowledge, the wavelength of 4496 nm with strong absorption in silica-based fibres is the longest emission wavelength from a CW fibre laser, and the span of 686 nm is also the largest tuning range achieved to date for any CW fibre laser. By further reducing the HCF transmission loss, increasing the pump power, improving the coupling efficiency, and optimizing the fibre length together with the pressure, the laser efficiency and output power are expected to increase significantly. This work opens new opportunities for broadly tunable high-power mid-IR fibre lasers, especially beyond 4 μm.

## Introduction

**Fig. 1:**
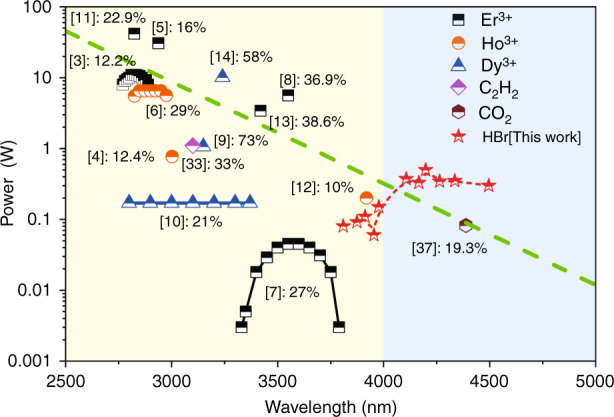
Summary of the state-of-art CW mid-IR fibre lasers in terms of output power and lasing wavelengths. The solid lines with symbols represent the continuously tunable output while the dotted line with symbols in this work represents the step-tunable output. The green dashed line is the fit of the data except for tunable results. The exponential decrease in output power with the emission wavelength is mainly due to the increase in the quantum defect between the pump and laser photon energies at longer wavelengths. The percentage close to the reference numbers refers to the corresponding slope efficiency. The red star symbols are the results in this work and two background light colours are employed to distinguish the wavelength boundary of 4 μm

Laser sources operating at the mid-infrared (mid-IR) are of considerable interest due to wide applications in biomedicine, spectroscopy, defence and manufacturing fields. Among these laser sources, fibre lasers have been favoured in recent years owing to their diffraction-limited beam quality, high conversion efficiency, long interaction length and compact system configuration^1,2^. However, due to the high phonon energy at approximately 1100 cm^−1^, silica-based fibres suffer from strong material absorption in the mid-IR range above 2.2 μm^1^. Extending the emission wavelength coverage of fibre lasers further into the mid-IR region, especially above 3 μm, is still an ongoing challenge^1^. For longer wavelengths, we usually use soft glass fibres of different host materials, including fluoride, telluride and chalcogenide glasses that have higher transparency in the mid-IR. A variety of transitions offered by rare-earth-doped soft glasses have been demonstrated for continuous-wave (CW) mid-IR fibre laser emission^3–14^. Figure 1 shows a summary of state-of-art CW mid-IR fibre output laser sources in terms of output power and slope efficiency as a function of lasing wavelengths. All the reported CW mid-IR lasers based on rare-earth-doped fibres operate below 4 μm thus far, and the output power decreases exponentially with the increasing emission wavelength, which is mainly due to the increase in the quantum defect between the pump and laser photon energies at longer wavelengths^1^. The solid lines with symbols show the tunable mid-IR fibre lasers. The tunable range of the fibre lasers is limited by the gain bandwidth of the doped ions. For mid-IR fibre lasers, the largest tunable range of 573 nm (2807–3380 nm) was obtained based on dysprosium-doped ZBLAN fibres with a maximum output power of 170 mW^10^. Recently, a pulsed laser operating at 5.38 μm was reported in terbium doped chalcogenide glass fibre^15^. Due to the high loss of the fibre, the laser threshold was just reached, and only emission spectrum was recorded without power values. In terms of mechanical strength, chemical durability, thermal conductivity, and optical nonlinearity, the performance of systems in the mid-IR based on these soft glasses is well behind that at shorter wavelengths using silica^1^.

Compared to silica-based solid core fibre lasers with difficulties in extending emission wavelength into the mid-IR spectral region, silica-based HCF gas lasers have opened new avenues for mid-IR light source. The mid-IR absorption loss of silica host materials in HCF can be reduced substantially owing to the small overlap of the core modes with the silica material, and the field in the silica material is at least an order of magnitude smaller than the peak field in the core, giving a higher damage threshold. The fast development of silica-based HCFs with low attenuation at mid-IR wavelengths^16–19^ is a significant motivation to develop mid-IR gas-filled HCF lasers which combine the advantages of both traditional fibre lasers and gas lasers^20–22^. In addition, the HCF can provide an ideal circumstance for the interaction of gas and light with a much longer interaction distance than traditional gas cells^23–25^. To date, several mid-IR lasers based on gas-filled HCFs have been demonstrated, which can be divided into two categories according to the operating mechanism, namely stimulated Raman scattering (usually using H_2_ and CH_4_^26–29^) and population inversion realized by intrinsic absorption of gas molecules (usually using C_2_H_2_, CO, N_2_O, HCN and CO_2_^30–37^). The laser threshold based on stimulated Raman scattering is about 5–6 orders of magnitude higher than that based on population inversion. Therefore, all the reported mid-IR Raman lasers based on gas-filled HCFs are pulsed^26–29^, while lasers based on population inversion are easier to realize CW mid-IR output^32–37^. Recently, a pulsed 4.6 μm laser based on N_2_O-filled Kagome HCFs was demonstrated^30^. However, limited by the transmission loss at the mid-IR, as well as the linewidth of the pump optical parametric oscillator, the laser slope efficiency is <3%. We recently reported a CW fibre laser at 4.3 μm by CO_2_- filled HCFs^37^, however, the possible output laser wavelength range is small (4.28–4.42 μm) due to the transition properties of CO_2_ molecules. In many applications, such as multispecies trace gas detection, IR countermeasures and free-space optical communications, a mid-IR fibre laser covering much wider wavelength range over 3–5 μm is required. The HBr gas medium has sparse rotational-vibrational energy level and relaxation characteristics, enabling the achievement of broadly tunable mid-IR lasers. Up to now, although traditional HBr gas lasers operating at mid-IR spectral region have been demonstrated in gas cells^38–42^. The effective interaction length of the gas media and the laser beam is very short, and the systems are usually bulky and cumbersome, seriously limiting the applications of these lasers. Therefore, the silica-based HCF with low mid-IR loss and long interaction length is envisioned to provide an ideal waveguide for the HBr mid-IR emission lines.

Here, we report the first CW HBr laser in a silica-based HCF. To demonstrate the widely wavelength tunable characteristics, a narrow linewidth 2 μm thulium-doped fibre amplifier (TDFA) seeded by a group of fine-tunable diode lasers is used to pump a 5 m-long anti-resonant HCF filled with low-pressure HBr gas. The wavelengths of the selected diode lasers cover the absorption lines of R(11), R(7), R(5), R(3), R(2) and R(0) for both isotopes H^79^Br and H^81^Br. A total of eleven narrow linewidth laser transitions, with five R-branch and six P-branch transitions, covering 3810–4496 nm are observed, which is the broadest tuning range and the 4496 nm output with strong absorption in silica-based fibres is the longest-wavelength among CW fibre lasers to the best of our knowledge. When the HBr pressure is 5 mbar, the maximum laser output of 500 mW at approximately 4.2 µm is achieved when pumped by the R(3) absorption line, with a slope efficiency of 18%. In addition, the output spectral components can be efficiently controlled under appropriate conditions and the laser exhibits excellent beam quality performance in the 4 μm CW region, with a measured beam quality factor *M*^2^ of approximately 1.2. This work paves the way for compact widely tunable high-power mid-IR fibre laser sources in the future.

## Results

### Theoretical analysis of the energy level transitions of the HBr molecule

**Fig. 2:**
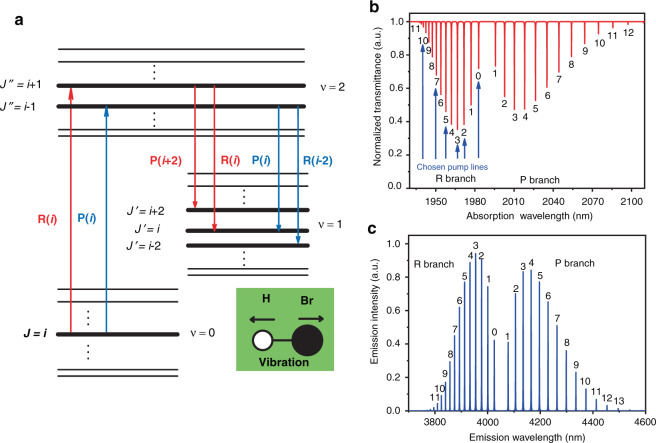
Characteristics of the radiative transition of the HBr molecule. **a** Schematic energy level diagram depicting a first-order rotational–vibrational overtone absorption transition and the corresponding laser transitions; inset: the vibrational normal mode of HBr. The small, white-filled circle represents the hydrogen atom, and the larger, black-filled circle represents the bromine atom; the horizontal line between them represents the electrostatic force, and the black arrows indicate the relative motions of the atoms. **b** Calculated absorption spectrum of H^79^Br molecules in the 2 μm spectral region from the HITRAN database^43^. The rotational lines become closer together with increasing rotational quantum number on the R-branch side, while the P-branch lines become more widely spaced as the rotational quantum number increases. The rotational lines marked with blue arrows represent the chosen pump wavelengths. **c** Corresponding emission spectrum of H^79^Br molecules in the 4 μm spectral region

The HBr molecule occurs in two isotopes, H^79^Br and H^81^Br, with almost equal natural abundances of 50.678 and 49.306%^43^, respectively. The corresponding isotope energy level mismatch between the two isotopes is approximately 50 GHz^39^. In addition, HBr is a diatomic molecule and consequently has only one vibrational normal mode, displayed in the inset of Fig. 2a. The vibrational normal mode can be expressed by vibrational quantum number *v*, which takes integer values from zero upwards. In each vibrational state, there is a series of rotational states due to molecular rotation, which are expressed by the rotational quantum number *J*. Owing to the small moments of inertia and strong molecular bonds, the HBr molecules have characteristically sparse rotational–vibrational energy levels^38,39^. The energy diagram of HBr energy levels for 4 μm emission is shown in Fig. 2a. The HBr molecules can be excited from the ground state (*v* = 0 vibrational state) to the upper level (*v* = 2 vibrational state) through rotational–vibrational transitions. Due to the large vibrational level spacing, the population of the lower laser level (*v* = 1 vibrational state) at room temperature is nearly zero^40^. Then, the HBr molecules can leave the upper level through radiation transition to the essentially empty *v* = 1 vibrational state, from which lasing occurs according to the selection rule ∆*J* = +1 (or ∆*J* = −1), referred to as R-branch (or P-branch) transitions. The population of the *v* = 1 vibrational state is transferred back to the vibrational ground state via vibrational relaxation (non-radiative transition) induced by intermolecular collisions.

From the HITRAN database^43^, Fig. 2b illustrates the first overtone absorption band of H^79^Br molecules at approximately 2 μm from the ground vibrational state *v* = 0 to the excited vibrational state *v* = 2. The number at each line represents the corresponding absorption lines of the R- or P-branch. The absorption intensity of different lines is directly proportional to the population of the rotational levels, which is governed by the Boltzmann distribution and the possibility of degeneracy^44^, indicating that the population rises to a maximum and then diminishes, as shown in Fig. 2b. The corresponding emission band is illustrated in Fig. 2c, which is similar to the absorption band. Table 1 summarizes the specific absorption and corresponding emission wavelengths of the R-branch. Owing to the increasing spacing between adjacent rotational states, a small tunable range of the pump, for example, from 1939 to 1983 nm, can generate an ultrabroad range of lasing wavelengths from 3760 to 4540 nm. In this experiment, the R(11), R(7), R(5), R(3), R(2) and R(0) absorption lines are chosen as pump wavelengths to achieve a tunable output lasing spectrum. Indeed, due to the strong absorption in the HCF, the relatively weak pump lines can also be absorbed by increasing the fibre length, so the potential lasing wavelength range can be broader. In addition, rotational relaxation usually occurs with a pulsed pump source with a long pulse duration due to inelastic collision between molecules, which will cause the population of the upper level to redistribute to other rotational levels and can also be used to achieve broadly tunable HBr lasers in the pulsed region^41^.

**Table. 1:** Calculated R-branch absorption lines and corresponding lasing lines of H^79^Br.

Pump lines	Pump λ (nm)	R-branch laser lines	Laser λ (nm)	P-branch laser lines	Laser λ (nm)
R(12)	1939.00	R(12)	3759.78	P(14)	4539.72
R(11)	1940.53	R(11)	3809.67	P(13)	4495.85
R(10)	1942.44	R(10)	3824.48	P(12)	4453.52
R(9)	1944.74	R(9)	3840.21	P(11)	4412.69
R(8)	1947.42	R(8)	3856.88	P(10)	4373.31
R(7)	1950.49	R(7)	3874.49	P(9)	4335.34
R(6)	1953.95	R(6)	3893.06	P(8)	4298.73
R(5)	1957.79	R(5)	3912.60	P(7)	4263.46
R(4)	1962.02	R(4)	3933.13	P(6)	4229.49
R(3)	1966.65	R(3)	3954.66	P(5)	4196.78
R(2)	1971.67	R(2)	3977.21	P(4)	4165.31
R(1)	1977.09	R(1)	4000.80	P(3)	4135.04
R(0)	1982.90	R(0)	4025.44	P(2)	4105.94

This shows the potential ultrabroad mid-IR wavelength range (>750 nm) of the HBr laser achieved by using a small range (~40 nm) tunable 2 μm pump source.

### Experimental layout and properties of the used HCFs and narrow linewidth pump source

**Fig. 3:**
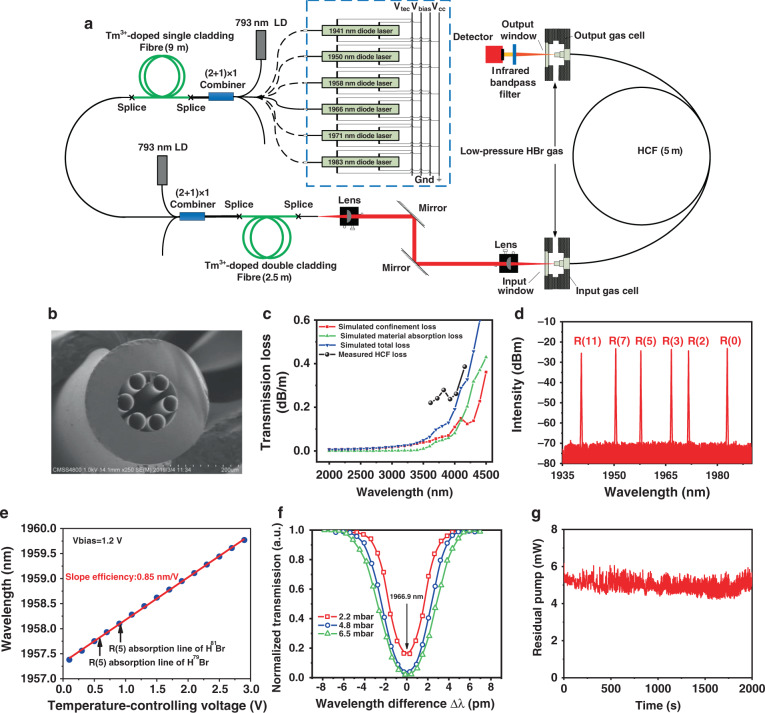
Experimental layout and measured and calculated properties of the HCF and the homemade pump source. **a** Experimental layout for 4 μm laser generation. **b** SEM images of the cross section of the HCF. **c** Comparison between simulated and measured loss of the HCF. **d** Measured spectrum of the pump system seeded by chosen absorption line wavelengths. **e** Output wavelength of the pump system changing with the Vtec of the R(5) diode laser. **f** Measured absorption line shapes of HBr under pressures of 2.2, 4.8 and 6.5 mbar seeded by the R(3) absorption line. **g** Variation in the measured residual pump power with time when tuned to the centre of the R(3) absorption line

The experimental layout is shown in Fig. 3a. High gain resulting from tight confinement of the pump light together with the active gas in the HCF permits to operate the laser in a single pass-configuration, which is actually the process of amplified spontaneous emission (ASE), without the use of any external resonator structure. The setup centres around a 5 m length of an in-house fabricated anti-resonant HCF surrounded by a single-ring 6-tube-cladding fused silica-based HCF structure, and a scanning electron micrograph (SEM) of the HCF cross-section is shown in Fig. 3b. The core diameter of the HCF is approximately 80 μm, the average inner diameter of the cladding capillary tubes is approximately 38 μm, and the average wall thickness is approximately 700 nm. This design can guide light over a large wavelength range from 1.9 to 4.5 μm, covering both the pump and corresponding laser wavelengths. Figure 3c shows the simulated HCF confinement loss and material absorption loss using a finite-element solver with a perfectly matched layer^45^, plotted in red and green curves respectively while the blue curve implies the total simulated loss including both two losses. The black dots illustrate the transmission loss value from 0.2 to 0.4 dB/m obtained using the cut-back measurements (see “Methods”), which are much higher than the simulated loss in shorter wavelength below 3.9 μm while close to the simulated loss in longer wavelength. The higher measured loss may be contributed by the structural nonuniformity in the longitudinal and transverse directions. The variation tendency of the measured HCF loss is consistent with that of simulated loss. The pump light is collimated passing through the first lens and aligned by two parallel silver-coated mirrors. Then the pump light is focused into the input end of the HCF through the second lens with a coupling efficiency of ~60%. At the output, an IR bandpass filter (>80% transmission at 4 μm and <0.1% transmission at 2 μm) is used to separate the generated mid-IR laser from the unabsorbed fraction of pump light that is transmitted by the HCF filled with HBr gas. The filtered output can be monitored using a thermal power metre or an optical spectrum analyzer (OSA). As displayed in Fig. 3a, the pump system is a TDFA with a two-stage amplifier arrangement seeded by one of six diode lasers corresponding to the R(11), R(7), R(5), R(3), R(2) and R(0) absorption lines of HBr which are marked in blue dashed line box. All diode lasers are driven by four voltage pins, namely Vtec (the temperature-controlling voltage), Vbias (the bias voltage), Vcc (the supply voltage) and Gnd (the ground connection). Each diode laser can be precisely tuned in a few nanometres by adjusting the Vbias and Vtec, covering the absorption line of HBr. Figure 3d shows the measured spectrum of the pump system seeded by different absorption line wavelengths, which has a very weak ASE background. Even with increasing output power of the pump system seeded by the R(5) absorption line to the maximum output power of 8 W, the ASE background is not obviously enhanced, illustrating that the output power is mainly concentrated at the central wavelength. Figure 3e shows the wavelength of the R(5) absorption line-seeded pump system varying with the Vtec when the Vbias is 1.2 V. The wavelength can be accurately tuned from 1957.4 to 1959.8 nm with a good linear relationship of 0.85 nm/V, covering the R(5) absorption line of both the H^79^Br isotope at 1957.8 nm and H^81^Br isotope at 1958.1 nm. The pump system seeded by the other wavelength diode lasers has similar wavelength tuning results (see the Supplementary Information).

For a gas laser, the linewidth and wavelength stability of the pump source are very important due to the narrow absorption linewidth of molecules, which is usually several hundred MHz at low gas pressure^21^, determined by collisional broadening and Doppler broadening. By precisely tuning the pump wavelength across the R(3) absorption line based on wavelength tuning results and then measuring the power transmitted by the HCF filled with different HBr gas pressures at each wavelength, the absorption linewidth can be measured (see “Methods”), as shown in Fig. 3f (the absorption linewidth of the other absorption lines is shown in the Supplementary Information). The discrete points are the measured data, and the curve is the corresponding smooth fitting curve. The three curves correspond to pressures of 2.2, 4.8 and 6.5 mbar with 3.6 pm (270 MHz), 4.8 pm (360 MHz) and 5.6 pm (420 MHz) absorption linewidths (full-width at half-maximum, FWHM), respectively. The spectral linewidth of the pump system is measured using a scanning Fabry–Perot (F–P) interferometer^46^ (setup shown in the Supplementary Information). When seeded by 1971 nm diode laser, the measured pump linewidth (FWHM) Δ*ν* is approximately 23 MHz, which is much narrower than the absorption linewidth of HBr. As long as the pressure is high enough, the pump power can be efficiently absorbed by HBr gas. Additionally, the wavelength stability of the pump system can be measured by monitoring the fluctuation of the residual pump power at the output end when the wavelength is tuned to the centre of the R(3) absorption line, where the transmitted pump power should be the minimum, as shown in Fig. 3f. The results for an approximately 30 min duration are shown in Fig. 3g. The residual pump power remains stable at approximately 5 mW, showing a very good wavelength stability of <1 pm, which is estimated from Fig. 3f, g.

### Measured widely step-tunable mid-IR spectra

**Fig. 4:**
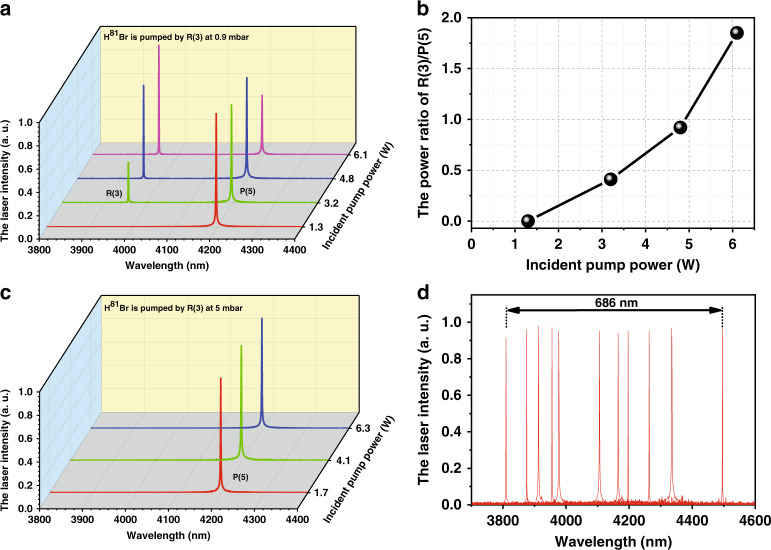
Measured widely step-tunable mid-IR emission spectra. **a** Measured output spectra changing with incident pump power at 0.9 mbar when pumped at the R(3) absorption line. **b** Intensity ratio of the R(3) to P(5) lines changing with incident pump power. The P-branch laser line can be observed first at a lower incident pump power owing to the larger cross-section than the R-branch laser line. **c** Measured output spectra changing with incident pump power at 5 mbar when pumped at the R(3) absorption line. **d** Measured output spectra of the HBr-filled HCF laser pumped at different absorption lines. From left to right are the R(11), R(7), R(5), R(3), R(2), P(2), P(4), P(5), P(7), P(9) and P(13) lasing transition lines

In the experiment, the measured mid-IR spectra are closely related to the HBr pressure and pump power. When pumped by any absorption line wavelength, both R-branch and P-branch laser lines should have been observed from the same upper level according to the Δ*j* = ±1 transitions, as explained in relation to Fig. 2a. As the R-branch and P-branch laser lines both share a common upper level, they compete with each other^32^. However, the intermolecular collisions are enhanced with increasing HBr pressure, leading to the rotational relaxation. Then the accumulated population of the upper level will transfer to other rotational energy level, causing other laser line emissions, which will be explained in the next section. Figure 4a shows the evolution of the laser peak intensity of the R(3) and P(5) laser transitions with respect to the incident pump power when the H^81^Br isotope gas is pumped by the 1966 nm R(3) absorption line at lower pressure of 0.9 mbar. The concrete peak intensity ratio of R(3) to P(5) is displayed in Fig. 4b. The peak intensity ratio of R(3) to P(5) increases from 0 to 1.8 with increasing incident pump power. Due to the larger Einstein A coefficient of the P-branch transition^32^, a single P-branch transition line might be observed first. Specifically, with increasing pump power, the population in the lower lasing level of the P(5) transition accumulates, causing a decrease in population inversion and reduced gain until it is exceeded by the gain provided to the competing line R(3) with a still empty lower lasing level, allowing this second line R(3) to lase as well. However, for a higher pressure of approximately 5 mbar which can be regarded as the transition pressure, the maximum 8 W power scaling of the pump system is not high enough to saturate the P-branch transition and the intermolecular collisions are not enhanced enough to cause the rotational relaxation. In this case, a pure spectrum with only the single laser line P(5) is observed while the R(3) laser transition has vanished owing to the lower pump power, as shown in Fig. 4c. By choosing appropriate HBr pressure that cannot give rise to rotational relaxation and pump power that is not high enough to allow R-branch transition to lase, the output spectral components can be efficiently controlled with a pure P-branch transition spectrum. Figure 4d shows all measured broadband step-tunable mid-IR emission spectra pumped with the six absorption line wavelengths in turn. A total of eleven laser transitions with five R-branch transitions and six P-branch transitions covering 3810–4496 nm are individually observed. The R(0) laser transition, which was expected at 4025 nm when pumped by the 1983 nm R(0) absorption line, is conspicuously absent due to the smallest emission cross section^43^.

### Energy level relaxation analysis

**Fig. 5:**
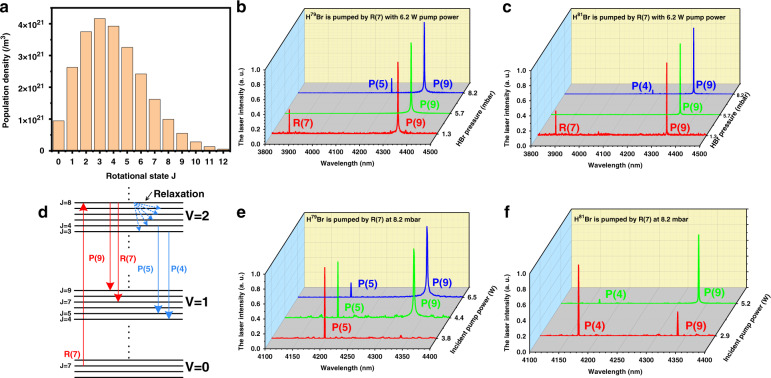
Energy level relaxation characteristics. **a** Boltzmann distribution in the *v* = 2 vibrational state. Measured output spectra changing with gas pressure when pumped at the R(7) line with 6.2 W pump power for the H^79^Br isotope (**b**) and H^81^Br isotope (**c**). **d** Schematic of population transition. The red lines show that the first overtone R(7) absorption results in fundamental R(7) and P(9) emissions. The blue lines show that the R-R relaxation results in the fundamental P(4) and P(5) transitions. **e**, **f** Measured output spectra changing with incident pump power at a gas pressure of 8.2 mbar when pumped at the R(7) line for the H^79^Br isotope (**e**) and H^81^Br isotope (**f**)

Relaxation caused by molecular collisions is a common process in gas lasers, leading to energy transfer between different states, including energy transfer between rotational states within the same vibrational state (R-R relaxation) and energy transfer between vibrational states (V-V relaxation)^47^. As the energy gap for R-R relaxation is smaller than that for V-V relaxation, the rate of R-R relaxation is two orders of magnitude larger than the rate of V-V relaxation^48^. The net population density transfer rate in R-R relaxation can be given by^47^:

1 $${{{\mathrm{A}}}}\left( J \right) = N_{{\rm{cp}}}\mathop {\sum}\limits_{J^\prime \ne J} {( - k_{J \to J^\prime }^{{\rm{ret}}}n_J + k_{J^\prime \to J}^{{\rm{ret}}}n_{J^\prime })}$$

where $k_{J \to J^\prime }^{{\rm{ret}}}$ is the rate constant of R-R relaxation for transfer from level *J* to *J*′ of the same vibrational state, *n*_*j*_ is the population density of the *J* rotational state, and *N*_cp_ is the density of possible collision partners. The first term on the right side describes the population transfer rate from the *J* rotational state to other rotational states, while the second term describes the opposite process. In thermal equilibrium without a laser field, the population obeys the Boltzmann distribution, and the R-R relaxation is in equilibrium with A(*J*) = 0. Therefore, the rate constant of R-R relaxation obeys the following formula:

2 $$k_{J^\prime \to J}^{{\rm{ret}}} = k_{J \to J^\prime }^{{\rm{ret}}}\frac{{2J + 1}}{{2J^\prime + 1}}\exp \left( {\frac{{E_{J^\prime } - E_J}}{{k_{\rm{B}}T}}} \right)$$

where *k*_B_ is the Boltzmann constant and *E*_*j*_ is the state energy of the *J* energy level. This formula also reveals that R-R relaxation will lead to a Boltzmann distribution for each rotational state of the upper vibrational state to achieve equilibrium, and the larger the density of possible collision partners is, the faster the equilibrium is reached. For the CW pump, there is sufficient time for the population to reach equilibrium. Therefore, regardless of which rotational state is pumped, before the signal laser is generated, the population in the upper vibrational state nearly obeys the Boltzmann distribution for each rotational state. Figure 5a shows the Boltzmann distribution in the upper vibrational state at 293 K (see Supplementary Information). The population in the *J* = 3 rotational state is dominant. Because the emission cross-sections of each P-branch emission line are relatively close, the gain of the signal emission line is mainly dependent on the population of the upper state. Therefore, in theory, the P(4) emission line is generated first under high pressure where the R-R relaxation is strong. Interestingly, in our experiment, the exact laser emission caused by R-R relaxation is related to the type of isotope. For H^79^Br, the P(5) emission line is dominant in the output spectra, while for H^81^Br, the P(4) emission line is dominant. The mechanism of the influence of the isotope on relaxation remains to be studied.

Figure 5b, c plots the output spectra changing with the gas pressure when the pump wavelength is tuned to the R(7) absorption line. The R(7) and P(9) transitions occur at a low gas pressure of 1.3 mbar, as similarly explained in relation to Fig. 4a. For a higher pressure of 5.7 mbar, disappearance of the R(7) signal occurs because the threshold of R(7) exceeds the maximum output power level of the pump system used in the present experiment, as the threshold increases with increasing pressure. With a further increase in gas pressure to 8.2 mbar, the P(4) or P(5) transition (corresponding to the H^81^Br or H^79^Br isotope, respectively) starts to occur owing to R-R relaxation. In addition, for R(2) or R(3) pumping, P(4) or P(5) corresponds not only to the targeted laser transition line but also to a relaxation transition line. In the experiment, only the corresponding targeted laser transition line without a relaxation transition line is observed under R(2) or R(3) pumping by changing the incident power. Figure 5e, f plots the output spectra changing with the incident pump power at a gas pressure of 8.2 mbar. With increasing incident pump power, the intensity of the P(4) or P(5) line gradually decreases, while the intensity of the P(9) line gradually increases. This is because for the P(9) transition, the upper state is the directly pumped state, but for the P(4) or P(5) transition, the population of the upper state comes from R-R relaxation (see Fig. 5d). Thus, the pump rate for the P(4) or P(5) transition is mainly determined by the R-R relaxation rate, while the pump rate for the P(9) transition is mainly determined by the pump power. Although under small-signal conditions, the gain of the P(4) or P(5) transition is larger than that of the P(9) transition, the gain of the P(4) or P(5) transition more easily reaches saturation due to the unchanged R-R relaxation rate, which inhibits the growth of the P(4) or P(5) laser power. However, for the P(9) transition, the stimulated emission is strengthened with increasing pump power. Therefore, in the competition between the P(9) transition and the P(4) or P(5) transition, the P(9) transition dominates with increasing incident power.

### Mid-IR laser power output properties

**Fig. 6:**
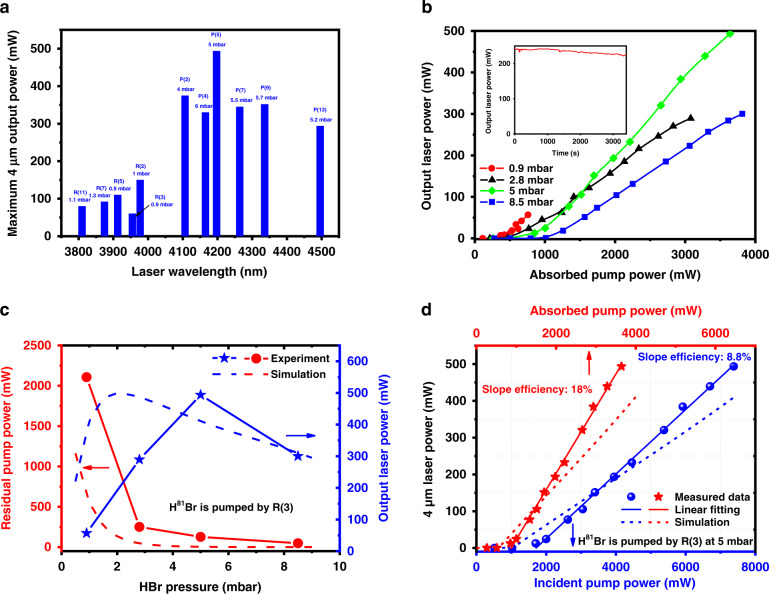
Measured mid-IR laser power properties. **a** Measured maximum output laser power of eleven laser transitions with appropriate pressure. **b** Measured output laser power at different HBr gas pressures as a function of the absorbed pump power when pumped by the R(3) absorption line of the H^81^Br isotope; inset: the stability of laser output with time, measured at 5 mbar in near 1 h. **c** Measured and simulated maximum output laser power and residual pump power as a function of HBr pressure when pumped by the R(3) absorption line at an incident pump power of approximately 7.5 W. Solid lines with points are measured data and dashed lines with points are simulated results. **d** Measured and simulated output laser power when pumped by the R(3) absorption line at the optimum pressure of 5 mbar as a function of incident pump power and absorbed pump power. The points are measured data. The solid lines are linear fitting of measured data and the dashed lines are simulated results

Since the two isotopes, H^79^Br and H^81^Br, gas molecules filled in the HCF with various pressures can be pumped by different absorption line wavelengths, a series of laser output power characteristics are measured. A simplified theoretical model is built to qualitatively analyze the laser generation process (see the Supplementary Information) and compare with the experimentally obtained results. Figure 6a illustrates the measured maximum output laser power of the individual 4 μm wavelength when H^79^Br and H^81^Br gas molecules are pumped. Due to the relatively low gain of the R-branch transition at approximately 4–6 mbar pressure (illustrated in Fig. 4c), only the P-branch transition laser occurs. In contrast, at approximately 0.9–1.3 mbar with higher incident pump power, the R-branch transition dominates over the whole output spectra, as explained in relation to Fig. 4a. Since the wavelength tuning range of the 1966 nm diode laser can only cover the R(3) absorption line of H^81^Br isotope molecules and the wavelength tuning range of the 1971 nm diode laser can only cover the R(2) absorption line of H^79^Br isotope molecules, R(11), R(7), R(5), R(2), P(2), P(4), P(7), P(9) and P(13) lasing transitions are measured when H^79^Br isotope molecules are pumped, while R(11), R(7), R(5), R(3), P(2), P(5), P(7), P(9) and P(13) lasing transitions are measured when H^81^Br isotope molecules are pumped. In addition, owing to the smallest emission cross-section, the R(0) lasing transition is absent. The maximum output of 500 mW is realized for the P(5) lasing transition when pumped by the R(3) absorption line under 5 mbar pressure. At lower pressure approximately 0.9–1.3 mbar, the pump power can just be partially absorbed with lower gain, which causes the measured maximum output laser power of R-branch transition is lower than that of P-branch transition. The measured output laser power at different HBr gas pressures as a function of the absorbed pump power when pumped by the R(3) absorption line is plotted in Fig. 6b (the output laser power when pumped by the other absorption lines is shown in the Supplementary Information). The 4 μm laser power increases with the absorbed pump power without saturation, reaching a maximum value of approximately 500 mW at 5 mbar. At a lower pressure of 0.9 mbar, the pump power is only partially absorbed, leading to a lower output. In addition, the lasing threshold, defined as the minimum absorbed pump power necessary to observe a 4 μm laser output, increases with the HBr pressure due to the enhanced intermolecular collisions. The inset in Fig. 6b illustrates the 1 h stability of the laser. The decline trend of the output laser is mainly owing to the ambient air leaking into the gas cells and HCF with time, decreasing the HBr gas purity and the output laser power. Especially for lower HBr gas pressure in the HCF, the leaky air will severely impair the output laser performance. Furthermore, Fig. 6c compares the measured and simulated maximum output laser power and residual pump power as a function of HBr pressure when pumped by the R(3) absorption line for an incident pump power of approximately 7.5 W. With increasing gas pressure, the increased molecular density in the HCF results in higher pump power absorption and gain, giving a higher laser output and a lower residual pump power. The output laser power reaches a maximum at an optimum pressure. However, beyond the optimum pressure, due to enhanced intermolecular collisions, the lifetime of the laser upper level is shortened, leading to reduced gain and low output power, although almost all pump power is absorbed. The differences between measured and simulated results occur because the actual pressure in HCFs remains imprecise and may be lower than the pressure showed in the vacuum gauge. Thus, higher molecular density in simulation model is considered compared with experimental data. The measured and simulated 4 μm laser power pumped by the R(3) absorption line as a function of incident pump power and absorbed pump power at the optimum pressure of 5 mbar is plotted in Fig. 6d. The measured output laser power increases almost linearly with the incident pump power and absorbed pump power beyond the threshold, reaching a maximum of 500 mW with slope efficiencies of 8.8 and 18% with respect to the incident pump power and absorbed pump power, respectively. Largely due to the discrepancy of pressures, the theoretical simulations are found to be in general agreement with the experimentally obtained data.

### Measurement of the mid-IR laser linewidth and beam quality factor *M*^2^

**Fig. 7:**
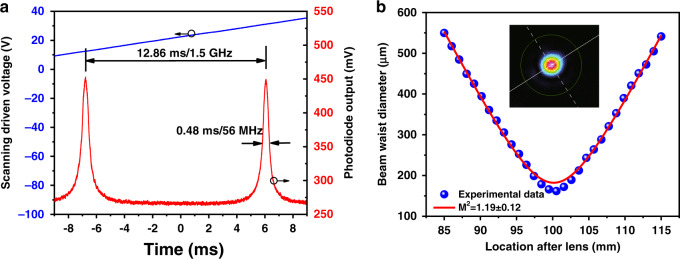
Measured linewidth and beam quality of the mid-IR laser. **a** Line shape of the output laser measured with a scanning F–P interferometer. **b** Typical output laser beam quality, where the inset shows the beam profile at the waist position in two dimensions

Both the linewidth and beam quality of the mid-IR laser are measured when the pump wavelength is tuned to the R(7) absorption line under 5.7 mbar pressure. Under such conditions, only the 4335 nm P(9) laser line is emitted, while the 3875 nm R(7) laser line is totally suppressed, as mentioned previously. Specifically, the linewidth of the molecular transition is determined by three broadening processes, namely, collisional broadening, Doppler broadening and negligible natural broadening. The total line shape is a Voigt profile, a convolution of a collisional line shape and a Doppler line shape. The line shape of the mid-IR laser is measured using a scanning F–P interferometer with a free spectral range (FSR) of 1.5 GHz (see “Methods”, setup shown in the Supplementary Information) and is shown in Fig. 7a. The time interval between the two peaks Δ*T* is approximately 12.86 ms, and the FWHM of one of the peaks Δ*t* is approximately 0.48 ms. Therefore, the linewidth of the mid-IR laser can be calculated by the formula (FSR/Δ*T*) × Δ*t*. The calculated linewidth is approximately 56 MHz, which is spectrally narrow without additional linewidth-limiting elements owing to the nature of gas molecule transitions. In addition, the output laser beam quality is characterized in terms of *M*^2^, which is determined by measuring the beam profiles of the 4 μm laser output at different longitudinal positions (see “Methods”). As illustrated in Fig. 7b, the output laser beam is observed to be near diffraction limited with an *M*^2^ of 1.19 ± 0.12. The inset shows the mode profile of the mid-IR laser beam at the waist position.

## Discussion

In summary, an optically pumped CW 4 μm HBr gas laser has been demonstrated in HCFs for the first time. By using a self-developed precisely tunable narrow linewidth TDFA as the pump source, eleven laser transitions covering 3810–4496 nm are individually observed in the HBr-filled HCF, with the longest emission wavelength and largest tuning range. The maximum output power of the mid-IR laser is 500 mW, with a slope efficiency of 18% with respect to the absorbed pump power when the H^81^Br isotope gas is pumped by the R(3) absorption line under 5 mbar pressure. The output mid-IR laser exhibits a narrow linewidth of approximately 56 MHz and a near diffraction-limited beam quality with an *M*^2^ of approximately 1.2. By further optimizing the fibre length and gas pressure, the efficiency and output power of the 4 μm laser are expected to be scaled significantly. In addition, widely tunable HBr lasers could be obtained by using a tunable 2 μm fibre laser covering many absorption lines.

Looking forward, several strategies are available to further improve the performance of mid-IR gas-filled HCF lasers. Since the pump light is usually coupled into HCFs by the method of spatial optical path coupling, the coupling efficiency is unstable and easily influenced by the external environment. One of the major development directions is the employment of all-fibre structure coupling with low loss between HCFs and solid-core fibres to replace the gas cell in the experimental setup and it includes directly fusion splice^49^, fibre tapered technology^50^, reverse-tapering method^51^ and so on. Achieving high power output is another important direction. To date, the highest power reported in gas-filled HCF lasers is only at the watt level. Several key issues need to be resolved for higher power output, mainly including a suitable narrow linewidth high-power pump source and low-loss coupling of a high-power pump laser. Obtaining more abundant laser wavelengths is also an important direction in the future. Compared with solid-core rare-earth-doped fibre lasers, gas gain media are more convenient to be replaced in gas fibre lasers, and there are more choices. If the HCF transmission bands are properly designed, then with suitable gases and pump sources, various laser wavelengths can be obtained, especially in the mid-IR region, which is not easy to achieve with traditional fibre lasers. The soft glass has been used to manufacture HCFs^52^, and far-IR gas-filled HCF lasers are expected to be realized. Additionally, gas-filled HCF lasers also have certain advantages in realizing laser output in the visible and ultraviolet bands. Especially in the ultraviolet band, the photon darkening effect is much weaker than that of solid-core fibres. The choice of gain media in the visible and ultraviolet bands is very rich, including common inert gases, various chlorides and metal vapours.

If the all-fibre coupling structure is employed to develop robust gas-filled HCF lasers with improved power level in the future, such mid-IR light source can provide more practical applications in lots of fields, such as remote monitoring of polluted gases or trace gases, free space communication in the atmosphere, infrared directional countermeasures, far-infrared frequency conversion, and so on.

## Methods

### HCF transmission loss measurement using cut-back methods

Cut-back methods are employed to experimentally measure the fibre transmission loss. Usually, a broadband light source is used to couple the light into the input end of the fibre, and the transmitted light at the output end of the fibre is recorded by an OSA before the cut-back. Then, a part of the fibre as far away from the fibre input end is cut off to avoid the effects of high order modes and the output spectrum is recorded again. Notably, the input to the fibre should be kept the same at all times and the fibre should be kept in a large loop (the loop diameter is always larger than 1 m in the experiment) to prevent the bending loss. In the experiment, we use an optical parametric oscillator (the wavelength can be individually tuned from 3 to 4.1 μm) to replace the broadband light source. A power metre is also used to replace the OSA to measure the output power at several wavelengths. The fibre attenuation is expressed as:

3 $$\alpha \left( \lambda \right) = \frac{1}{{{L}}} \times 10 \times {\rm{log}}\frac{{{{P}}_2(\lambda )}}{{{{P}}_1(\lambda )}}$$

in which *L* is the cut HCF length. *P*_1_(λ) is the output power of the original long HCF before cut-back, while *P*_2_(λ) is the output power of the remaining HCF after cut-back.

### Absorption line measurement

Each seed diode laser source of the pump system has four pins (shown in the experimental layout of Fig. 3a), in which Vcc is the fixed supply voltage (usually 5 V), Gnd represents the ground connection, Vtec is the temperature-controlling voltage (from 0.1 to 3 V), and Vbias is the bias voltage (from 0 to 1.4 V). Specifically, except that the wavelength tuning range of the 1966 nm diode laser can only cover the R(3) absorption line of the H^81^Br isotope molecule and the wavelength tuning range of the 1971 nm diode laser can only cover the R(2) absorption line of the H^79^Br isotope molecule, the wavelength tuning range of other four diode lasers can cover the corresponding absorption lines of both the H^79^Br and H^81^Br molecules. The output centre wavelength can be adjusted by Vtec and Vbias, and in the experiment, we usually set Vbias to 1.2 V. Figure 3e shows the centre wavelength of the R(5) absorption line as a function of Vtec with a good linear relationship. By adjusting the Vtec step by step to tune the pump wavelength across the absorption line and then measuring the output power at each single wavelength, the absorption linewidth can be measured and normalized.

### Mid-IR laser power and spectra measurement

As the experimental setup in Fig. 3a shows, the output 4 μm laser and the residual pump transmit through the output window (WG31050, Thorlabs), which has a transmission of 87% at both the 2 and 4 μm bands. An IR bandpass filter (FB4250-500 or FB4000-500, Thorlabs) is set at the output end of the system to filter out the residual pump. A thermal power metre or a spectrometer (OSA-207C, Thorlabs) is used to measure the mid-IR laser power or the spectra.

### Laser linewidth measurement

The mid-IR laser beam is aligned with the scanning F–P interferometer installed in the standard optical adjusting frame through two mirrors. Then, a lens is placed in front of the interferometer so that the beam waist is in the centre of the interferometer. The scanning voltage provided by the control box is connected to the oscilloscope while driving the piezoelectric ceramic (PZT) in the interferometer. The signal obtained by the photodiode detector in the interferometer is also connected to the oscilloscope after being amplified by the control box (setup shown in the Supplementary Information).

### Beam quality factor *M*^2^ measurement

The *M*^2^ reported here is measured using two plano-convex lenses of 40 and 100 mm focal lengths. The output end of the HCF is placed at the focal point of the first lens, collimating the mid-IR laser beam. The second lens is employed to focus the collimated beam. Then, a CCD camera installed on a translation stage can scan the beam profiles at different positions along the propagation axis. A criterion of 1/*e*^2^ of maximal intensity is applied to define the beam size at all measurement points and the value of *M*^2^ can be calculated.

## Supplementary information

Supplementary Information

## Data Availability

The data that support the results in this paper and other findings of this study are available from the corresponding author upon a reasonable request.
