# Supplementary material for: Towards high-power mid-IR light source tunable from 3.8 to 4.5 µm by HBr-filled hollow-core silica fibres
Source: Light Sci Appl. 2022 Jan 13;11:15. doi: 10.1038/s41377-021-00703-6 (PMC8755826; doi:10.1038/s41377-021-00703-6)
Supplement: Supplementary file 1 — Supplementary Information [file 41377_2021_703_MOESM1_ESM.docx]

**Supplementary Information for**

Towards high-power mid-IR light source tunable from 3.8 to 4.5 µm by HBr-filled hollow-core silica fibres

Zhiyue Zhou^1,2,3^, Zefeng Wang^1,2,3,*^, Wei Huang^1,2,3^, Yulong Cui^1,2,3^, Hao Li^1,2,3^, Meng Wang^1,2,3^, Xiaoming Xi^1,2,3^, Shoufei Gao^4^ and Yingying Wang^4^

^1^College of Advanced Interdisciplinary Studies, National University of Defense Technology, Changsha, 410073, China

^2^State Key Laboratory of Pulsed Power Laser Technology, Changsha, 410073, China

^3^Hunan Provincial Key Laboratory of High Energy Laser Technology, Changsha, 410073, China

^4^Institute of Photonics Technology, Jinan University, Guangzhou, 511443, China

^*^Email address: zefengwang_nudt@163.com

**S1. Simplified simulation model of CW HBr-filled HCF laser**

For the experimental results without the observation of signal lines caused by relaxation at lower pressure, a simplified theoretical model that does not consider R-R relaxation can be built to qualitatively analyse the laser generation process.


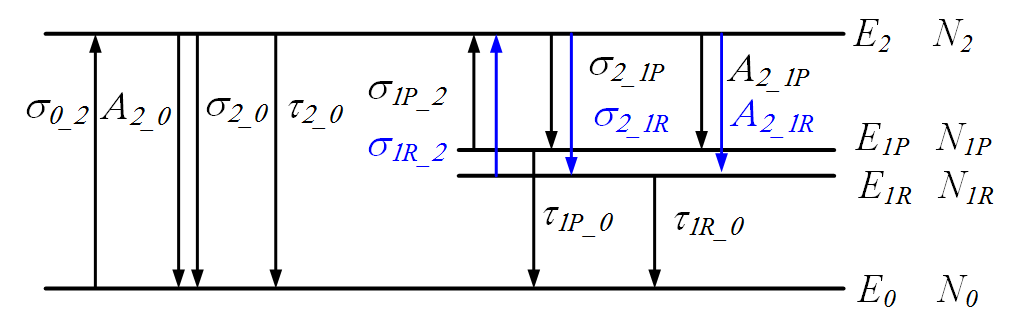


Fig. S1. Schematic of the level transitions in the simplified model.

Figure S1 shows a schematic of the energy level transitions considered in the model. *E_0_* is the ground state, and *E_2_* is the upper level. *E_1P_* and *E_1R_* are the targeted lower laser levels corresponding to P-branch and R-branch transitions, respectively. *N_2_*, *N_1P_*, *N_1R_* and *N_0_* are the population densities of the corresponding energy levels. *σ_0_2_*, *σ_1P_2_* and *σ_1R_2_* represent the simulated absorption cross sections from energy levels *E_0_*, *E_1P_*, and *E_1R_* to *E_2_*, respectively. *σ_2_0_*, *σ_2_1P_* and *σ_2_1R_* represent the corresponding simulated emission cross sections. *A_2_0_*, *A_2_1P_* and *A_2_1R_* represent the corresponding spontaneous transition probabilities. *τ_2_0_*, *τ_1P_0_* and *τ_1R_0_* represent the non-radiative transition lifetimes (or V-T relaxation lifetimes) from energy levels *E_2_*, *E_1P_*, and *E_1R_* to *E_0_*, respectively. Thus, the rate equation can be given in matrix form for brevity:

$\left[\begin{matrix} -\frac{I_{p}\sigma_{0\_2}}{h\upsilon_{p}} & \frac{1}{\tau_{1P\_0}} & \frac{1}{\tau_{1R\_0}} & \frac{I_{p}\sigma_{2\_0}}{h\upsilon_{p}}\text{+}\frac{1}{\tau_{2\_0}}\text{+}A_{2\_0} \\ 0 & -\frac{I_{sP}\sigma_{1P\_2}}{h\upsilon_{sP}}-\frac{1}{\tau_{1P\_0}} & 0 & \frac{I_{sP}\sigma_{2\_1P}}{h\upsilon_{sP}}+A_{2\_1P} \\ 0 & 0 & -\frac{I_{sR}\sigma_{1R\_2}}{h\upsilon_{sR}}-\frac{1}{\tau_{1R\_0}} & \frac{I_{sR}\sigma_{2\_1R}}{h\upsilon_{sR}}+A_{2\_1R} \\ 1 & 1 & 1 & 1 \end{matrix}\right]\left[\begin{matrix} N_{0} \\ N_{1P} \\ N_{1R} \\ N_{2} \end{matrix}\right]=\left[\begin{matrix} \frac{dN_{0}}{dt} \\ \frac{dN_{1P}}{dt} \\ \frac{dN_{1R}}{dt} \\ N_{tot} \end{matrix}\right]$ (1)

where *h* is the Planck constant. *v_p_*, *v_sP_* and *v_sR_* are the frequencies of the pump, P-branch and R-branch lasers, respectively. *I_p_*, *I_sP_* and *I_sR_* are the light intensities of the pump, P-branch and R-branch lasers, respectively. As mentioned previously, *σ* is the absorption or emission cross section. *A* is the Einstein A coefficient. *τ* is the lifetime of non-radiative transition. *σ*, *A* and *τ* are marked with a subscript to indicate the corresponding energy levels. The frequency and *A* coefficient (shown in Tab. 1) can be obtained from the HITRAN database^1^. The emission cross section is calculated by^2^:

$\sigma\text{=}\frac{A\lambda^{2}S\left(v\right)}{8\pi}$ (2)

where *S*(*v*) is the line shape function (Voigt profile). The linewidth of the gas molecular transition is mainly determined by collisional broadening and Doppler broadening. The collisional broadening is described by a Lorentzian line shape function^2^:

$S_{c}(v)=\frac{\Delta v_{c}/2\pi}{\left(v-v_{0}\right)^{2}+\left(\Delta v_{c}/2\right)^{2}}$ (3)

where *v_0_* is the resonance frequency and ∆*v_c_* is the linewidth of the Lorentzian line shape function. The linewidth ∆*v_c_* is equal to *αp*, where *α* is estimated 9.4 MHz Torr^-1^ for HBr gas and *p* is the gas pressure. The Doppler line shape function is given by^2^:

$S_{d}(v)=\frac{2}{\Delta v_{d}}(\frac{\ln2}{\pi})^{\frac{1}{2}}\exp(\frac{-4\ln2(v-v_{0})^{2}}{\Delta v_{d}^{2}})$ (4)

where ∆*v_d_* is the linewidth of the Doppler line shape function^2^:

$\Delta v_{d}\text{=}2v_{0}(\frac{2k_{B}T}{m}\ln2)^{\frac{1}{2}}$ (5)

where *k_B_* is the Boltzmann constant. *T* is the temperature and *m* is the mass of the molecule.

The line shape of the HBr transition is the result of a combination of collisional broadening and Doppler broadening, which is expressed in the form of a Voigt line shape. The Voigt line shape at the resonance frequency takes the following form^2^:

$S_{v}(v)=\frac{2}{\Delta v_{d}}\left(\frac{\ln2}{\pi}\right)^{\frac{1}{2}}erfcx\left(2\sqrt{\ln2}\frac{\Delta v_{c}}{\Delta v_{d}}\right)$ (6)

here, erfcx is the scaled complementary error function^2^.

Tab. 1 Transition parameters of the first overtone absorption transition and the corresponding second overtone transition

| Pump lines | Einstein A-coefficient (s^-1^) | Lower-state energy  (cm^-1^) | R-branch laser lines | Einstein A-coefficient (s^-1^) | Lower-state energy  (cm^-1^) | P-branch laser lines | Einstein A-coefficient (s^-1^) | Lower-state energy  (cm^-1^) |
| --- | --- | --- | --- | --- | --- | --- | --- | --- |
| R(12) | 0.155 | 1294.4 | R(12) | 5.403 | 3817.2 | P(14) | 8.083 | 4248.9 |
| R(11) | 0.154 | 1096.4 | R(11) | 5.527 | 3624.7 | P(13) | 8.107 | 4025.3 |
| R(10) | 0.154 | 914.5 | R(10) | 5.644 | 3447.9 | P(12) | 8.128 | 3817.2 |
| R(9) | 0.153 | 748.8 | R(9) | 5.752 | 3286.9 | P(11) | 8.146 | 3624.7 |
| R(8) | 0.152 | 599.5 | R(8) | 5.850 | 3141.7 | P(10) | 8.163 | 3447.9 |
| R(7) | 0.151 | 466.6 | R(7) | 5.935 | 3012.5 | P(9) | 8.182 | 3286.9 |
| R(6) | 0.150 | 350.1 | R(6) | 6.004 | 2899.3 | P(8) | 8.206 | 3141.7 |
| R(5) | 0.149 | 250.2 | R(5) | 6.050 | 2802.2 | P(7) | 8.241 | 3012.5 |
| R(4) | 0.147 | 166.9 | R(4) | 6.066 | 2721.2 | P(6) | 8.295 | 2899.3 |
| R(3) | 0.144 | 100.2 | R(3) | 6.031 | 2656.3 | P(5) | 8.388 | 2802.2 |
| R(2) | 0.140 | 50.1 | R(2) | 5.908 | 2607.6 | P(4) | 8.558 | 2721.2 |
| R(1) | 0.131 | 16.7 | R(1) | 5.596 | 2575.2 | P(3) | 8.906 | 2656.3 |
| R(0) | 0.110 | 0 | R(0) | 4.728 | 2558.9 | P(2) | 9.797 | 2607.6 |

The equations for the pump and signal laser corresponding to the propagation process are:

$\left\{\begin{matrix} \frac{dI_{p}}{dz}=-I_{p}\sigma_{0\_2}N_{0}+I_{p}\sigma_{2\_0}N_{2}-\alpha_{p}I_{p}+\Omega h\upsilon_{p}A_{2\_0}N_{2} \\ \frac{dI_{sP}}{dz}=-I_{sP}\sigma_{1P\_2}N_{1P}+I_{sP}\sigma_{2\_1P}N_{2}-\alpha_{sP}I_{sP}+\Omega h\upsilon_{sP}A_{2\_1P}N_{2} \\ \frac{dI_{sR}}{dz}=-I_{sR}\sigma_{1R\_2}N_{1R}+I_{sR}\sigma_{2\_1R}N_{2}-\alpha_{sR}I_{sR}+\Omega h\upsilon_{sR}A_{2\_1R}N_{2} \end{matrix}\right.$ (7)

where Ω is a factor that describes the fact that only a fraction of spontaneously emitted light is guided along the longitudinal direction of the HCF and is estimated approximately 10^-7^ here. *α_p_*, *α_sP_* and *α_sR_* are the fibre attenuation of the pump, P-branch and R-branch signal lasers, with values of 0.53 dB m^-1^, 0.3 dB m^-1^ and 0.3 dB m^-1^, respectively. For the HCF gas laser operating in the CW region, the population distribution is in the steady state with *dN_0_*/*dt*=0, *dN_1P_*/*dt*=0 and *dN_1R_*/*dt*=0. Therefore, formula (1) can be rewritten as:

$\left[\begin{matrix} -\frac{I_{p}\sigma_{0\_2}}{h\upsilon_{p}} & \frac{1}{\tau_{1P\_0}} & \frac{1}{\tau_{1R\_0}} & \frac{I_{p}\sigma_{2\_0}}{h\upsilon_{p}}\text{+}\frac{1}{\tau_{2\_0}}\text{+}A_{2\_0} \\ 0 & -\frac{I_{sP}\sigma_{1P\_2}}{h\upsilon_{sP}}-\frac{1}{\tau_{1P\_0}} & 0 & \frac{I_{sP}\sigma_{2\_1P}}{h\upsilon_{sP}}+A_{2\_1P} \\ 0 & 0 & -\frac{I_{sR}\sigma_{1R\_2}}{h\upsilon_{sR}}-\frac{1}{\tau_{1R\_0}} & \frac{I_{sR}\sigma_{2\_1R}}{h\upsilon_{sR}}+A_{2\_1R} \\ 1 & 1 & 1 & 1 \end{matrix}\right]\left[\begin{matrix} N_{0} \\ N_{1P} \\ N_{1R} \\ N_{2} \end{matrix}\right]=\left[\begin{matrix} 0 \\ 0 \\ 0 \\ N_{tot} \end{matrix}\right]$ (8)

By using the difference method to calculate formulas (7) and (8), we can obtain the simulated results for describing the experimental phenomena. In addition, the lifetimes *τ_1P_0_* and *τ_1R_0_* are equal to 1/(*k_v-v_N_tot_*), where *k_v-v_* describes the rate of non-radiative transition, which takes the value of approximately 10^-16^ m^3^ s^-1^, and *N_tot_* is the total population density of the gas, which is dependent on pressure. For lifetime *τ_2_0_*, *k_v-v_* is approximately 10^-17^ m^3^ s^-1^.


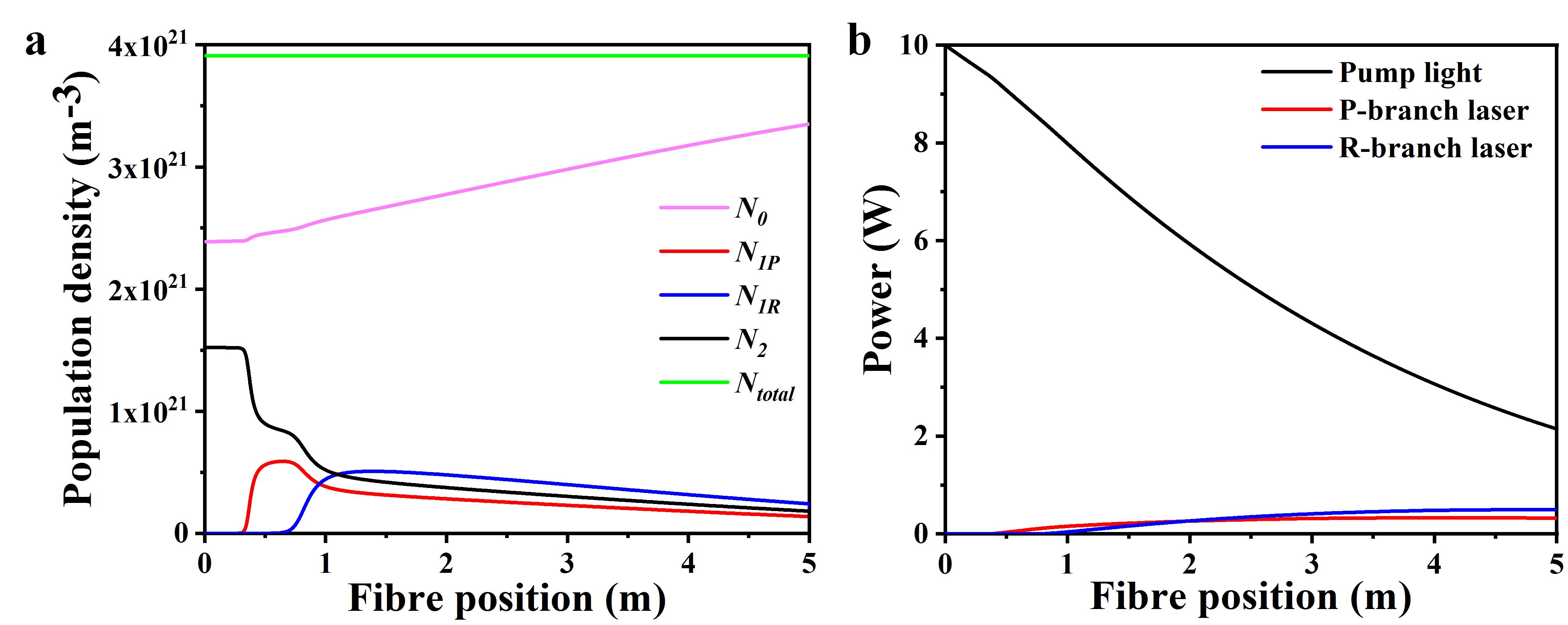


Fig. S2. **a**, Population distribution along the HCF at a pump power of 10 W. **b**, Power distribution along the HCF at a pump power of 10 W. All the simulated results are calculated under conditions with a pressure of 1 mbar, a temperature of 293 K and the absorption line of R(2) for the H^79^Br isotope.

Figure S2 shows the simulated population and power distribution along the HCF. Since the population distribution is in the steady state, the population density *N_2_* of the upper level *E_2_* has already been accumulated at the input end of the HCF, remaining unchanged until the threshold is reached. With the increasing propagation distance, the population density *N_1P_* increases first compared with the population density *N_1R_* owing to the larger Einstein A coefficient, as shown in Fig. S2a.The threshold of the P-branch laser is lower than that of the R-branch laser, but the growth rate of the R-branch laser is larger. Thus, in the HCF, the P-branch signal appears first, and at the output end of the HCF, the power of the R-branch signal already exceeds that of the P-branch signal, as shown in Fig. S2b.


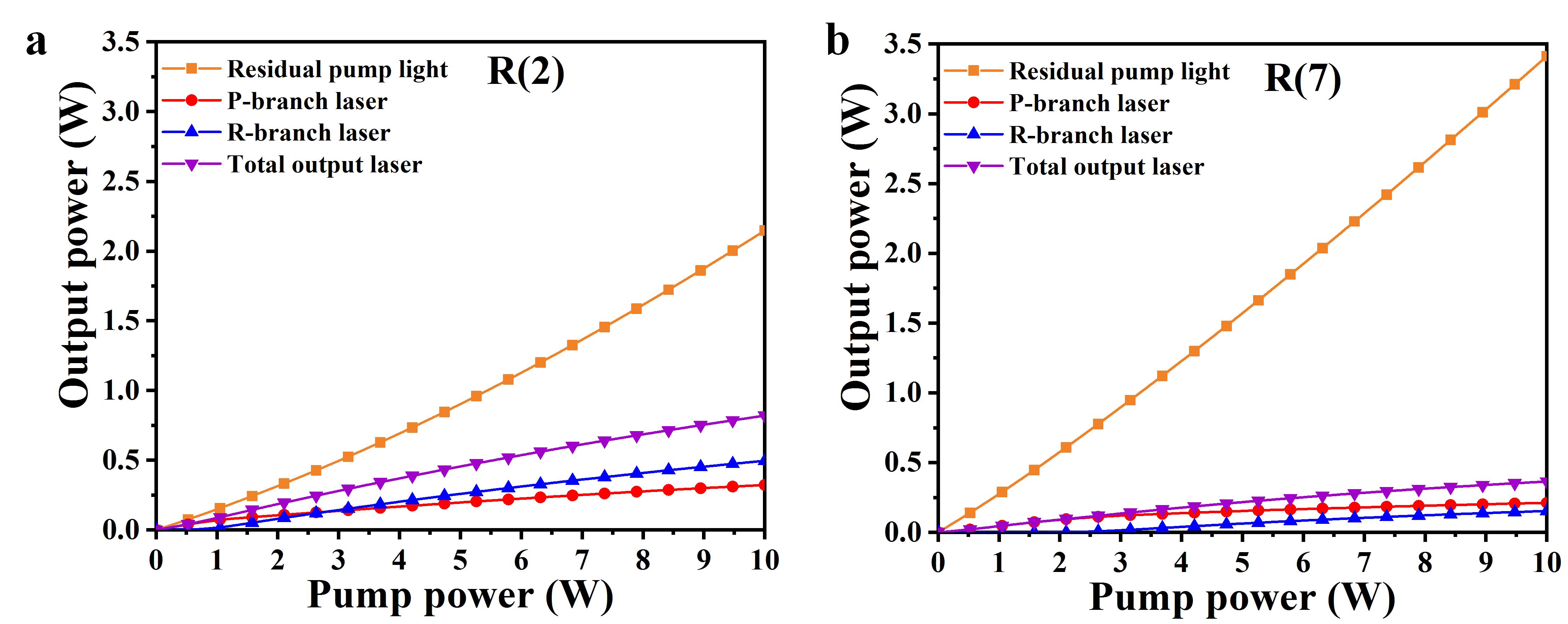


Fig. S3. **a**, **b**, Evolution of pump and signal power with the incident pump power at a pressure of 1 mbar when pumped at the R(2) absorption wavelength **a**, and R(7) absorption wavelength **b**.

The simulated laser power properties employing the theoretical model at the R(2) and R(7) pump wavelengths are illustrated in Fig. S3a and Fig. S3b respectively. For the R(7) pump wavelength, the absorption intensity of the pump power is lower than that for the R(2) pump wavelength. This is because in the simulation model, the excited population is dependent on the population of the rotational level in the ground state *E_0_*, while the population distribution in the ground state in thermal equilibrium obeys the Boltzmann distribution. Thus, the excited population for the R(2) pump is larger than the excited population for the R(7) pump, resulting in a difference in the absorption ability. In addition, from zero to the maximum pump power of 10 W, although the power of the R-branch signal is always lower than that of the P-branch signal for the R(7) pump wavelength. The tendency that the power of the R-branch signal would exceed that of the P-branch signal with further increasing pump power can be seen. At higher pump power above approximately 2.6 W for the R(2) pump wavelength, the output power of the R-branch signal is more than that of the P-branch signal.


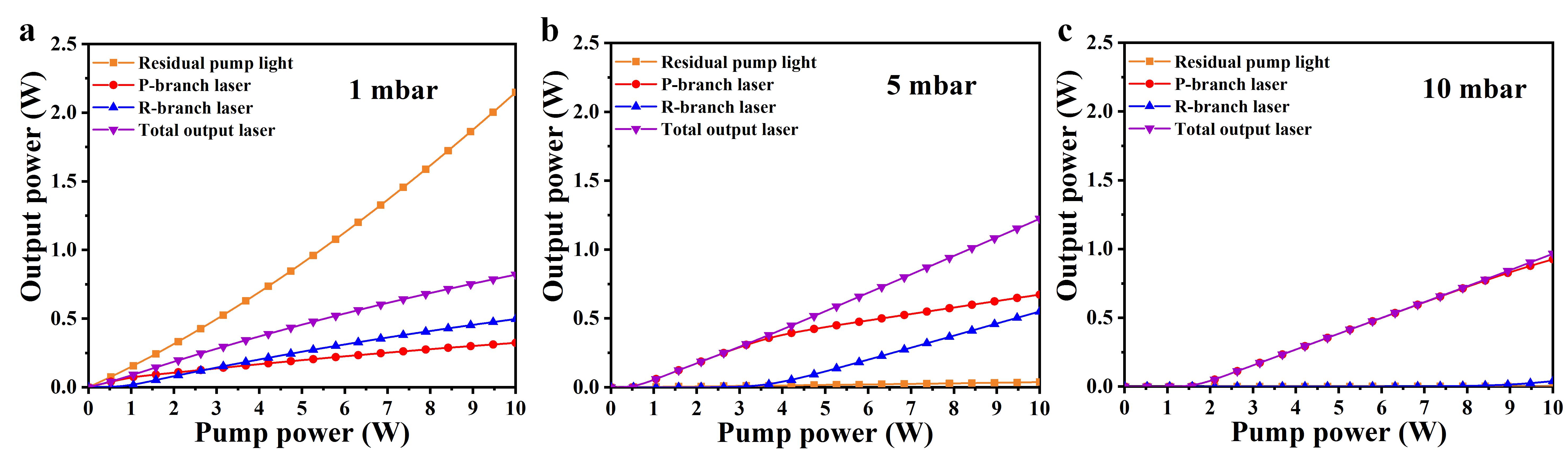


Fig. S4. **a**, **b**, **c**, Evolution of the pump and signal power with the incident pump power when pumped at the R(2) absorption wavelength at pressures of 1 mbar **a**, 5 mbar **b**, and 10 mbar **c**.

Figure S4 shows the simulated laser power properties with different gas pressures when pumped at the R(2) absorption wavelength. With increasing gas pressure, the absorption of the pump power increases. In addition, the thresholds of the signal also increase with increasing gas pressure. This is because the collision strengthening at higher pressure leads to a decrease in the non-radiative transition lifetime, which means an increase in loss. In addition, the degree of the increase in the threshold of the R-branch signal is much larger than that of the P-branch signal. Therefore, in the case of CW pump power, at high pressure, the R-branch signal will disappear. Notably, at high pressure, the R-R relaxation process is also strengthened, and the signals caused by relaxation can also be observed; thus, this simplified model cannot be used. However, the trend shown by the model will provide a good reference for gas-filled HCF lasers operating in CW regime.

**S2. Population distribution of the energy levels**

The absorption intensity of different lines directly depends on the population density of the rotational levels of the upper vibrational state. The calculation of the population distribution in thermal equilibrium of the upper vibrational state is based on the Boltzmann distribution and the degeneracy factor:

$n(v,J)=N_{tot}\frac{(2J+1)\exp(\frac{-E(v,J)}{k_{B}T})}{\sum_{v,J} \left(2J+1\right)\exp(\frac{-E(v,J)}{k_{B}T})}$ (9)

where *N_tot_* is the total population density of molecules, *n*(*v, J*) is the state population density, *E*(*v, J*) is the state energy, 2*J*+1 is the degeneracy factor of the rotational state, and *k_B_* is the Boltzmann constant. The temperature *T* is set to 293 K. Only the *v*=2 vibrational state and the rotational quantum number *J* from 1 to 12 are considered in the calculation. *N_tot_* can be given by *p*/*k_B_T* multiplied by the ratio of the isotope. Figure S5 shows the calculated results, indicating that the population rises to a maximum at the *J*=3 rotational state and then decreases.


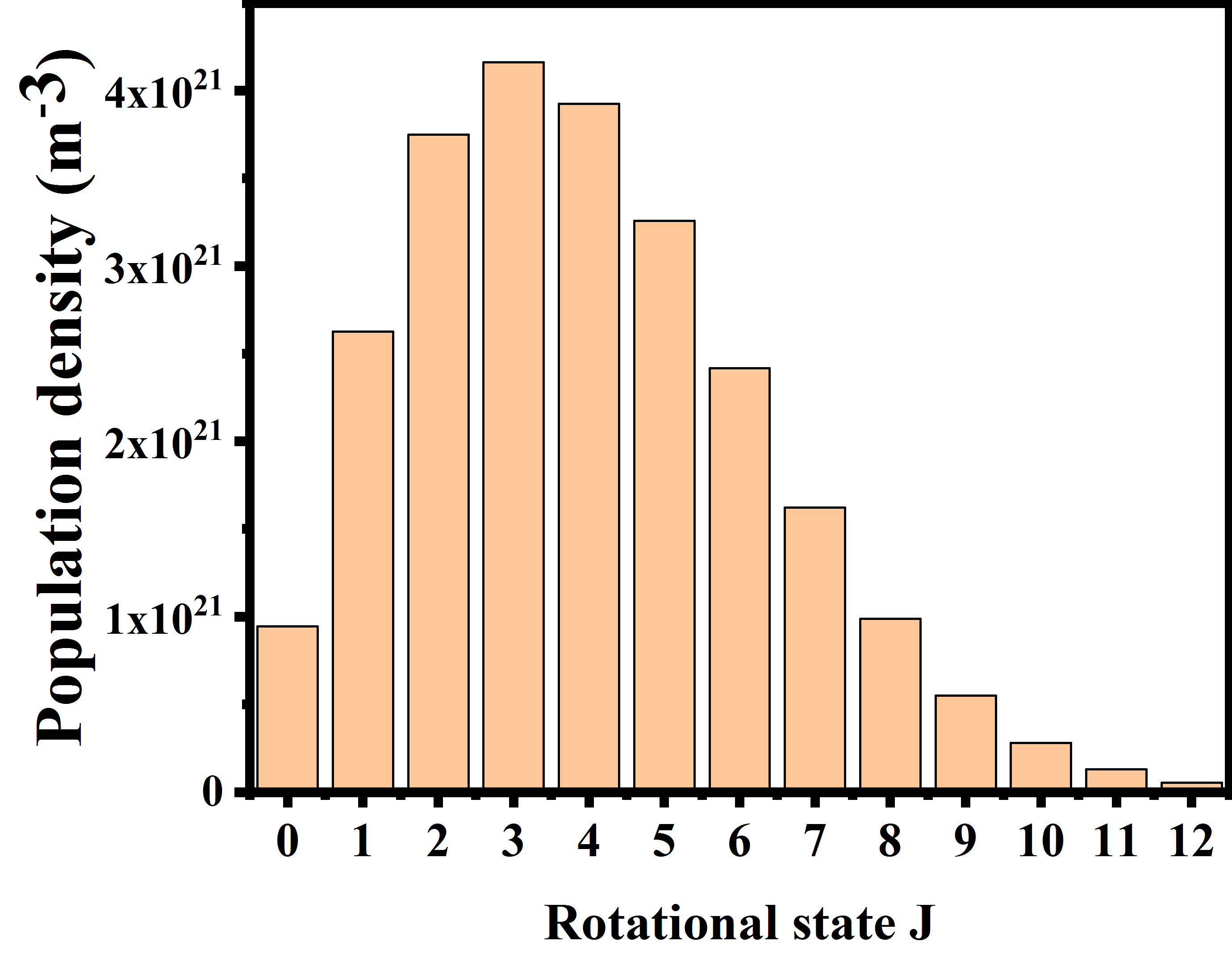


Fig. S5. Population distribution in the *v*=2 vibrational state

**S3. Spectra of the pump system seeded by the R(7), R(5), R(3) and R(2) absorption line wavelengths with increasing pump power**

With increasing output power of the pump system from 1 W to the maximum output power of 8 W, the spectra seeded by the R(7), R(5), R(3) and R(2) absorption line wavelengths are measured, as shown in Fig. S6. All measured spectra remain nearly unchanged, indicating that the ASE background of the pump spectra is not obviously enhanced and the output power of the pump system is mainly concentrated at the central wavelength.


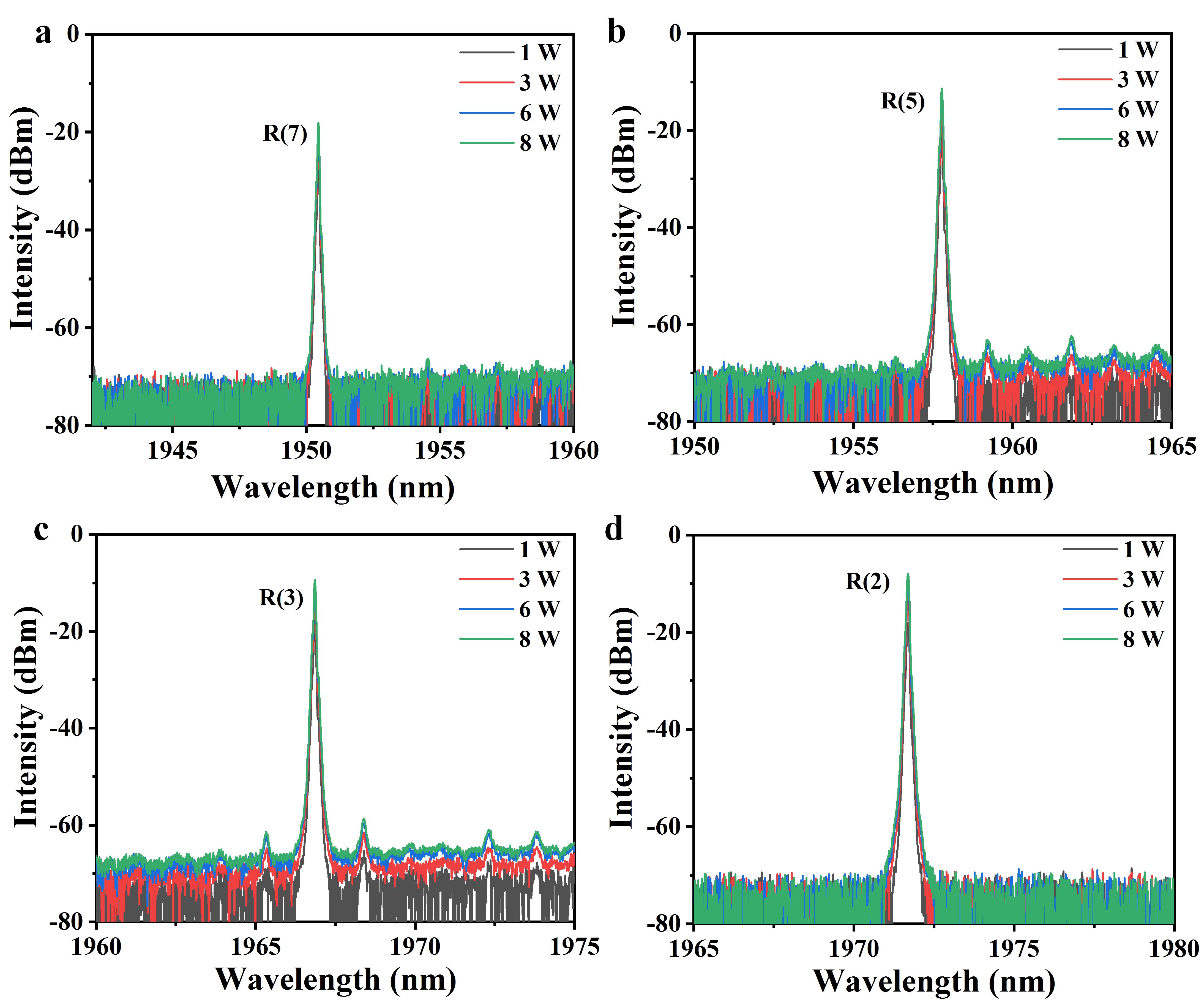


Fig. S6. **a**, **b**, **c**, **d**, Measured spectra of the pump system seeded by the 1950 nm R(7) absorption line **a**, 1958 nm R(5) absorption line **b**, 1966 nm R(3) absorption line **c** and 1971 nm R(2) absorption line **d**. The black, red, blue and green lines correspond to the spectra measured at 1 W, 3 W, 6 W and 8 W output power of the pump system, respectively.

**S4. Wavelength tuning results of the pump system seeded by the R(7) and R(2) absorption line wavelengths**

Each seed diode laser source of the pump system has four pins (shown in the experimental layout), in which Vcc is the fixed supply voltage (usually 5 V), Gnd represents the ground connection, Vtec is the temperature control voltage (from 0.1 V to 3 V), and Vbias is the bias voltage (from 0 V to 1.4 V). The output centre wavelength can be adjusted by Vtec and Vbias, and in the experiment, we usually set Vbias to 1.2 V. Figure S7 shows the centre wavelength of the R(7) and R(2) absorption lines as a function of Vtec, with a good linear relationship, which can be used to measure the absorption linewidth of HBr gas filled in the HCF. The discrete points are the measured data, and the curve is the corresponding fitting curve. Specifically, the pump system corresponding to 1950 nm R(7) can be tuned from 1949.6 nm to 1951.9 nm with a 0.8 nm V^-1^ slope, covering the corresponding absorption lines of both the H^79^Br and H^81^Br molecules. The pump system corresponding to 1971 nm R(2) can be tuned from 1971.4 nm to 1971.8 nm with a 0.38 nm V^-1^ slope, only covering the absorption line of the H^79^Br molecule.


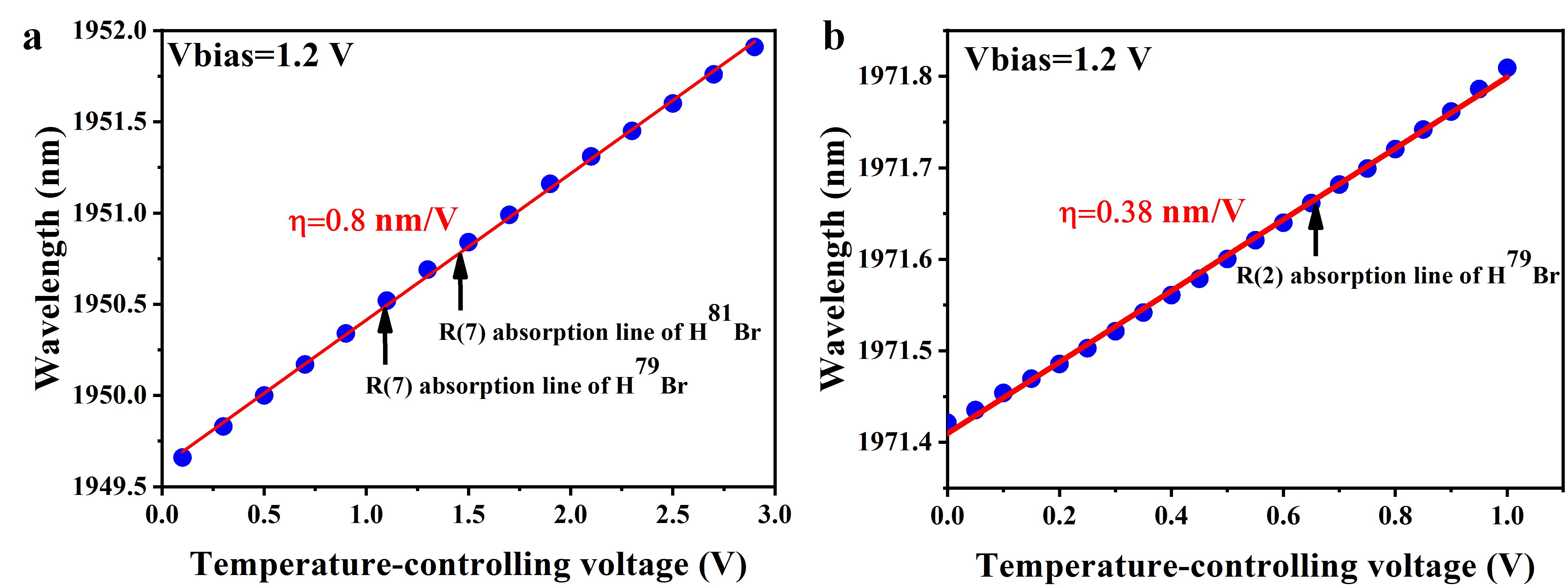


Fig. S7. **a**, **b**, Centre wavelength of the pump system seeded by the 1950 nm R(7) absorption line **a** and 1971 nm R(2) absorption line **b** with respect to the temperature-controlling voltage.

**S5. Linewidth measuring experimental setup and absorption linewidth of the R(7), R(5), R(2) and R(0) absorption lines**

A diagram of the measurement of the pump light is shown in Fig. S8a (and the mid-IR laser linewidth is measured using the same experimental setup). Through two mirrors and two plano-convex lenses, the pump laser seeded by 1971 nm R(2) absorption line is collimated, focused and then coupled into the F-P interferometer. The scanning voltage provided by the control box is connected to the oscilloscope while driving the piezoelectric ceramic (PZT) in the F-P interferometer. The signal obtained by the photodiode detector in the F-P interferometer is also connected to the oscilloscope after being amplified by the control box. The output of the oscilloscope is shown in Fig. S8b. In a scanning voltage cycle of the F-P cavity, two peaks are obtained, and the interval between them is Δ*T* = 15.56 ms; an enlarged picture of the second pulse is inserted in the middle. The full width at half maximum (FWHM) of the peak Δ*t* is approximately 236 μs, and then, the laser linewidth can be given by:

$FWHM\text{=}(\frac{FSR}{\Delta T})\Delta t$ (10)

where FSR is the free spectral range, which is 1.5 GHz used in the experiment. Therefore the measured linewidth of the pump system is approximately 23 MHz.

Due to the relationship of the centre wavelength and voltage displayed in Fig. S7, the absorption linewidth of the R(7), R(5), R(2) and R(0) absorption line shapes can be measured by precisely tuning the pump wavelength across the absorption line and then measuring the power transmitted by the HCF filled with different HBr gas pressures at each single wavelength, as shown in Fig. S8c-i. Since the wavelength tuning range of the 1971 nm diode laser can only cover the R(2) absorption line of the H^79^Br isotope molecule, the line shape of the R(2) absorption line of the H^81^Br isotope molecule is absent.


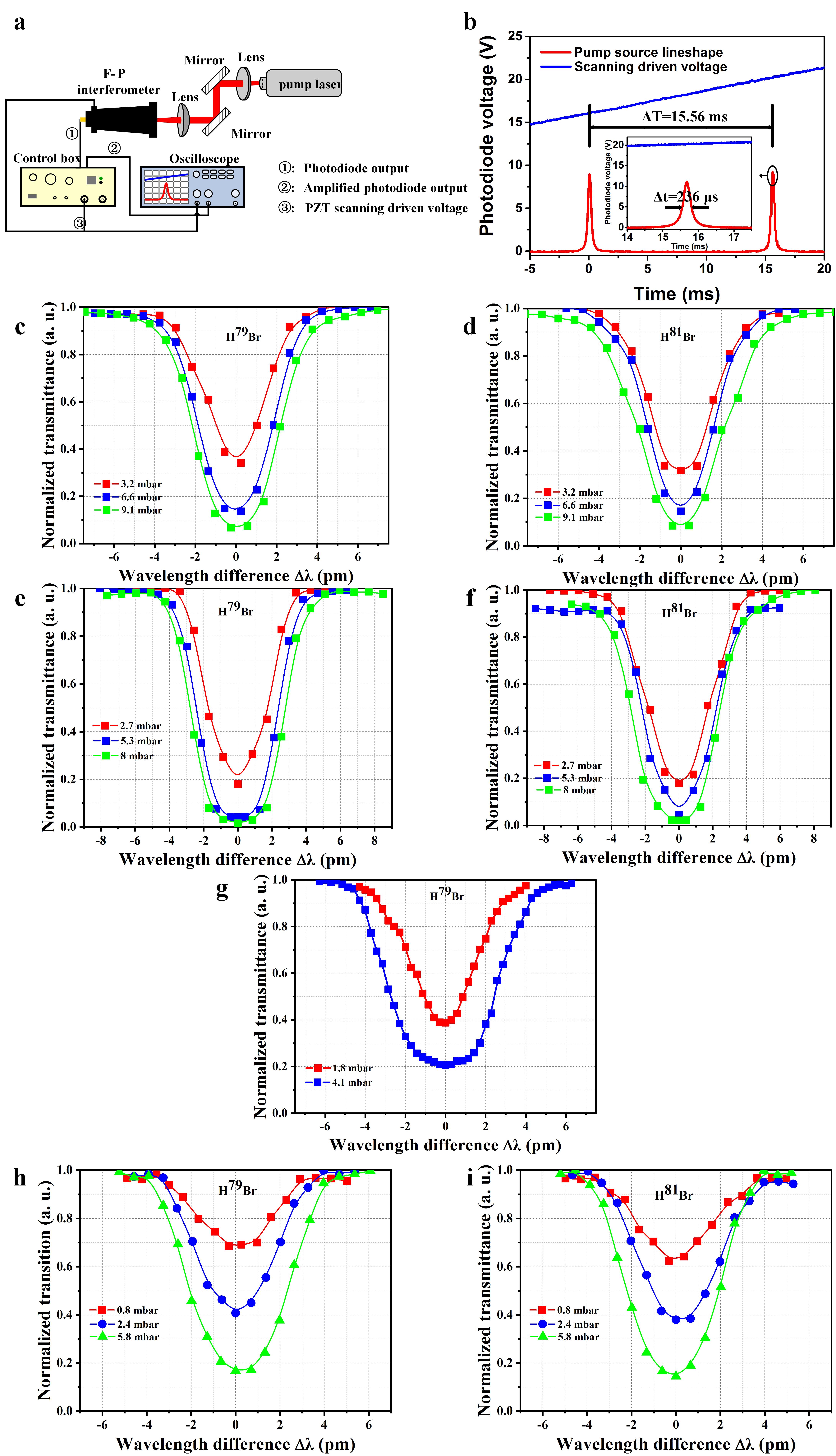


Fig. S8. **a** Experimental setup for the measurement of the pump laser linewidth by an F-P interferometer. **b** Measured results of the pump linewidth. Measured absorption line shapes of H^79^Br isotope gas using the R(7) absorption line **c**, H^81^Br isotope gas using the R(7) absorption line **d**, H^79^Br isotope gas using the R(5) absorption line **e**, H^81^Br isotope gas using the R(5) absorption line **f**, H^79^Br isotope gas using the R(2) absorption line **g**, H^79^Br isotope gas using the R(0) absorption line **h**, and H^81^Br isotope gas using the R(0) absorption line **i**.

**S6. Watt-level mid-infrared laser output with respect to absorbed pump power when two isotope, H^79^Br and H^81^Br, gas molecules filled in the HCF are pumped by the R(7), R(5), R(2) and R(0) absorption line wavelengths**

In addition to measured output laser power at different HBr gas pressures when pumped by the R(3) absorption line,a series of mid-IR laser output power characteristics pumped by the R(7), R(5), R(2) and R(0) absorption line wavelengths are also measured, as plotted in Fig. S9.


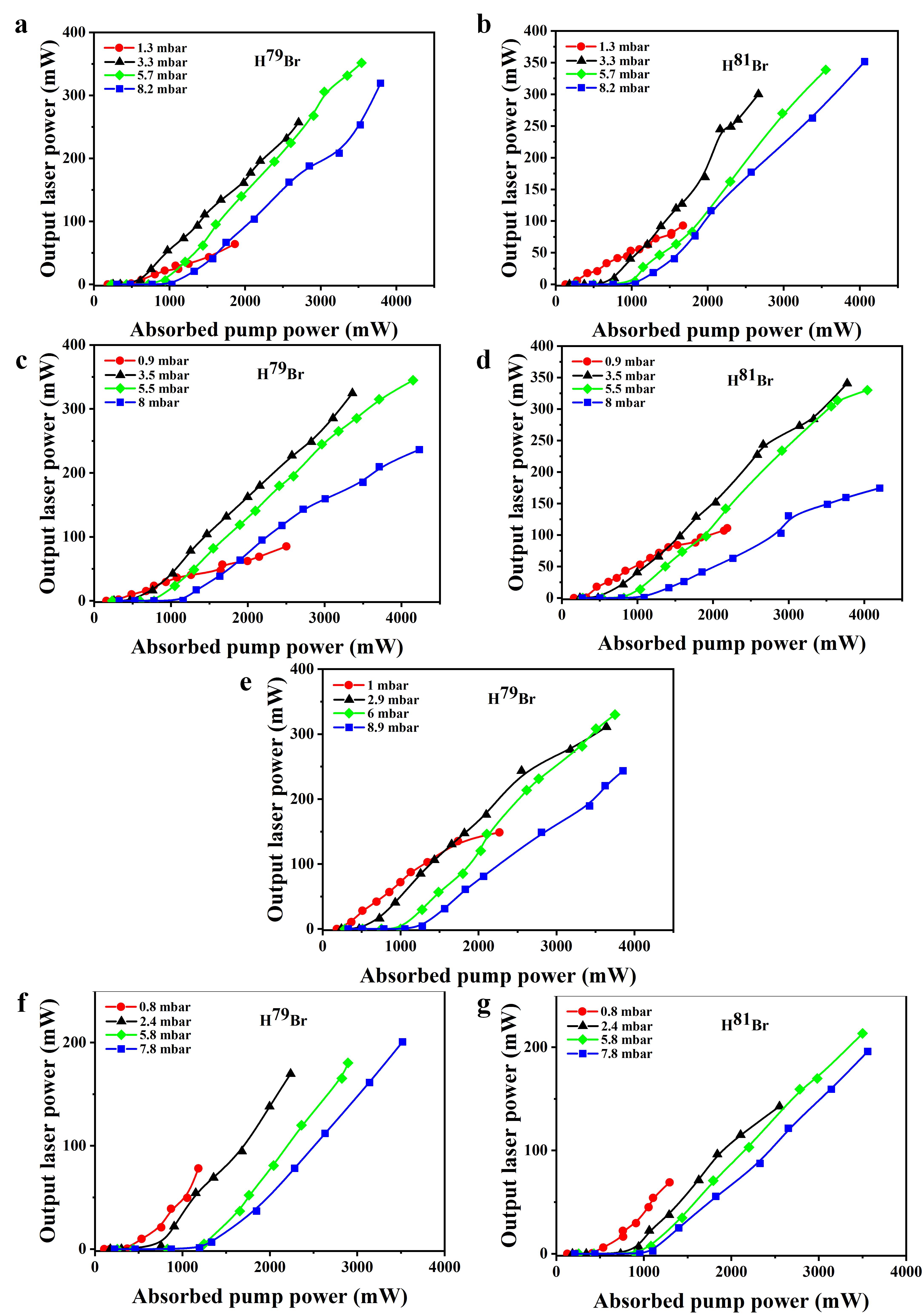


Fig. S9. **a**, **b**, **c**, **d**, **e**, **f**, **g**, Measured output laser power at different HBr gas pressures as a function of the absorbed pump power when pumped by the R(7) absorption line of the H^79^Br isotope **a**, R(7) absorption line of the H^81^Br isotope **b**, R(5) absorption line of the H^79^Br isotope **c**, R(5) absorption line of the H^81^Br isotope **d**, R(2) absorption line of the H^79^Br isotope **e**, R(0) absorption line of the H^79^Br isotope **f**, and R(0) absorption line of the H^81^Br isotope **g**.

**Reference:**

1 HITRAN spectroscopic database. https://hitran.iao.ru/bands/simlaunch?mol=16.

2 A. Ratanavis, Theoretical and experimental studies of optically pumped molecular gas lasers, Ph.D. thesis, the University of New Mexico, Albuquerque, New Mexico (2010).
